# Differential replication dynamics for large and small *Vibrio *chromosomes affect gene dosage, expression and location

**DOI:** 10.1186/1471-2164-9-559

**Published:** 2008-11-26

**Authors:** Rikard Dryselius, Kaori Izutsu, Takeshi Honda, Tetsuya Iida

**Affiliations:** 1 Laboratory of Genomic Research on Pathogenic Bacteria, Research Institute for Microbial Diseases, Osaka University, 3-1 Yamadaoka, Suita, Osaka 565-0871, Japan; 2 Department of Bacterial Infections, Research Institute for Microbial Diseases, Osaka University, 3-1 Yamadaoka, Suita, Osaka 565-0871, Japan

## Abstract

**Background:**

Replication of bacterial chromosomes increases copy numbers of genes located near origins of replication relative to genes located near termini. Such differential gene dosage depends on replication rate, doubling time and chromosome size. Although little explored, differential gene dosage may influence both gene expression and location. For vibrios, a diverse family of fast growing gammaproteobacteria, gene dosage may be particularly important as they harbor two chromosomes of different size.

**Results:**

Here we examined replication dynamics and gene dosage effects for the separate chromosomes of three *Vibrio *species. We also investigated locations for specific gene types within the genome. The results showed consistently larger gene dosage differences for the large chromosome which also initiated replication long before the small. Accordingly, large chromosome gene expression levels were generally higher and showed an influence from gene dosage. This was reflected by a higher abundance of growth essential and growth contributing genes of which many locate near the origin of replication. In contrast, small chromosome gene expression levels were low and appeared independent of gene dosage. Also, species specific genes are highly abundant and an over-representation of genes involved in transcription could explain its gene dosage independent expression.

**Conclusion:**

Here we establish a link between replication dynamics and differential gene dosage on one hand and gene expression levels and the location of specific gene types on the other. For vibrios, this relationship appears connected to a polarisation of genetic content between its chromosomes, which may both contribute to and be enhanced by an improved adaptive capacity.

## Background

Vibrios constitute a broad family of gammaproteobacteria with over 100 members classified (NCBI taxonomy browser). They are ubiquitous within marine and estuarine environments and the ecological roles for individual species are diverse. A common characteristic, however, is their ability to adapt and survive within various niches either as free-swimmers or in symbiotic or pathogenic association with diverse aquatic organisms such as plankton, coral, fish and shellfish. Moreover, several *Vibrio *species are capable of infecting humans with *Vibrio cholerae*, *V. parahaemolyticus *and *V. vulnificus *as the most common causes of disease [1,2].

A shared trait among vibrios is the presence of two unequally sized chromosomes [3]. The larger shows a more constant size, a lower interspecies sequence variability and harbour many of the genes involved in essential biosynthetic pathways while the smaller *Vibrio *chromosome is highly variable in size and contains relatively more species specific and unclassified genes [3-6]. This unusual structure and the distinct distribution of genetic content between the replicons has prompted studies on how the system is maintained and has also initiated discussion about fitness benefits with a divided genome [4,6,7].

Regarding the maintenance issue, most knowledge about chromosomal replication and partitioning has been gained from studies on *V. cholerae*. For example, it has been shown that the two chromosomes display different segregation patterns [8-10] and utilise separate sets of partition proteins [11,12]. It has further been demonstrated that while the large chromosome origin of replication is similar to *oriC *of *Escherichia coli*, the small bears resemblance to those of certain plasmids [13]. Nevertheless, the number of initiations for the two replicons remains equal and strictly follows the cell cycle [14]. Therefore, as the difference in size between the replicons theoretically results in differing replication times it was suggested that initiation of replication is synchronised to maintain an equal number of small and large chromosomes [14]. However, more recent studies indicate that inter-chromosomal synchrony between the *V. cholerae *chromosomes likely occurs at the level of termination [15,16].

Bacteria with divided genomes must overcome additional obstacles to accurately distribute genetic material to daughter cells, yet the evolutionary success of the broad and diverse *Vibrio *family implies that split genomes may be beneficial. A possible advantage is that multiple replicons allow faster replication which in turn could lead to faster growth rates [17]. This view is supported by the fact that several *Vibrio *species display unusually short multiplication times [18,19]. Another potential benefit may be that multiple chromosomes provide the means to regulate gene expression in a replicon-wide manner by alterations in the 1:1 balance between copy numbers [11]. Such regulation could facilitate large scale adaptations in response to changes in growth conditions [13], for example when the bacterium associates with or dissociates from a host organism. Considering that the genetic content differs for the small and large *Vibrio *chromosomes and that the two chromosomes utilise partly different mechanisms for initiation of replication [13] and partitioning [12], this is not an unlikely assumption. Consistent with this idea, over-expression of the distinct large or small chromosome replication initiator protein of *V. cholerae *results in over-initiation of the respective chromosome they control [20]. Also, *V. cholerae *cells harboring unequal numbers of small and large chromosomes were recently obtained by deleting the small chromosome specific partitioning genes [12]. Although these studies reveal technical possibilities for alterations in chromosome balance, no wild type vibrio with differing numbers of large and small chromosomes has yet been detected.

Interestingly, vibrios may possess an intrinsic mechanism to differentiate gene copy numbers between the chromosomes through gene dosage associated with replication [15,21]. Such gene dosage occurs as replication always initiates at an origin and proceeds in a bidirectional manner towards the terminus of replication. This mode of replication means that genes located near the origin are duplicated earlier than other genes, which enables higher expression. Therefore, with a given replication speed, there are two main factors that influence gene dosage; initiation rate and replicon size. A higher initiation rate results in an increased average difference in copy numbers between origin proximate and terminus proximate genes as replication takes up a larger proportion of the cell cycle. For the same reason, gene dosage differences are more pronounced for a larger over a smaller replicon assuming equal initiation frequencies [21].

Although an influence from gene dosage on bacterial gene expression levels has only occasionally been reported [22-24], it is assumed to affect gene positioning [25]. In line with this, altered gene dosage has been used as an explanation for decreased fitness and sometimes deleterious effects that can follow chromosomal rearrangements [26-28] as such events simultaneously change the distance to the origin of replication and thereby average copy numbers for a large number of genes. The impact from gene dosage has also been examined at the scale of genomic conservation, and highly expressed genes tend to locate near the origin of replication, especially for bacteria with fast cell division rates [21,29]. Furthermore, it has been reported that a high level of gene dosage correlates with a higher degree of genomic stability as fast multiplying bacteria display a stronger conservation of gene positioning [21]. Therefore, despite the fact that direct experimental evidence is scarce, these reports give support for gene dosage as an important factor in the evolution of bacterial genomes, especially for fast growing species.

In the case of vibrios, which have their genomes distributed between two unequally sized chromosomes and also display very short multiplication times, differential gene dosage could have a strong impact. The short multiplication times would result in large gene dosage differences within each chromosome. Also, assuming an equal replication speed for the two replicons, size differences would lead to different gene dosage between the chromosomes. Indeed, a study employing flow cytometry in combination with computer modelling revealed convincing experimental evidence for increased gene dosage differences within the large than within the small chromosome of actively growing *V. cholerae *[15]. In addition, this examination showed growth rate dependent variations in relative gene copy numbers both within and between the chromosomes and that differing timings of replication initiation creates overall highest copy numbers for genes located near the origin of replication of the large chromosome. A later study employing fluorescence microscopy to detect relative abundances of large and small chromosome origins of replication confirmed these enhanced and growth rate dependent gene dosage differences for the large chromosome relative to the small [16]. However, this report also indicated some question marks regarding an earlier replication start for the large chromosome, at least under certain growth conditions.

In an attempt to extend current knowledge about vibrio replication dynamics and gain insight into how this affect expression and genetic distribution, we here employed real-time PCR to quantify relative abundances of origin and terminus proximate DNA for both chromosomes of actively growing *V. parahaemolyticus*, *V. cholerae *and *V. vulnificu*s. For *V. parahaemolyticus*, microarray analyses at both the genomic and transcription levels were also conducted. We further examined the location of distinct gene types within five sequenced and annotated *Vibrionaceae *genomes and related this to replication and expression patterns.

## Results

### Estimates of large and small chromosome origin/terminus ratios based on doubling times

To examine replication dynamics and gene dosage effects for the two *Vibrio *chromosomes we first aimed to establish appropriate growth conditions. As larger inter- and intra-chromosomal gene dosage differences should be obtained from quickly multiplying cells, we determined doubling times for three *Vibrio *species (*V. parahaemolyticus*, *V. cholerae *and *V. vulnificus*, see Table 1) incubated in rich media at 37°C. As comparisons, we determined doubling times of *V. parahaemolyticus *grown in rich broth at 20°C, which more closely resembles the natural growth temperature for vibrios, and in minimal broth at 37°C. The results are summarised in Table 2 and show shortest doubling times for *V. parahaemolyticus *(12–14 min) followed by *V. cholerae *(16–20 min) and *V. vulnificus *(18–22 min). Doubling times were tripled for *V. parahaemolyticus *incubated at a lower temperature (36–42 min) and quadrupled for cells grown in minimal broth (50–60 min).

**Table 1:** Strain designations, purpose of use and chromosome sizes for bacteria employed in this study

**Strain designation**	Purpose*	**Size large chr (Mb)**	**Size small chr (Mb)**	**Ref**
*Vibrio parahaemolyticus *RIMD 2210633	Exp/Comp	3.29	1.88	[37]
*Vibrio cholerae *El Tor Inaba RIMD 2203577	Exp	2.85	1.20	-
*Vibrio vulnificus *ATCC27562	Exp	3.40	1.75	-
*Vibrio cholerae *O1 El Tor N16961	Comp	2.96	1.07	[11]
*Vibrio vulnificus *YJ016	Comp	3.35	1.86	[38]
*Vibrio fischeri *ES114	Comp	2.91	1.33	[39]
*Photobacterium profundum *SS9	Comp	4.09	2.24	[40]

*Experimental and/or computational analyses.

**Table 2:** Doubling times, replication times and theoretical origin/terminus ratios for *V. parahaemolyticus*, *V. cholerae *and *V. vulnificus*

**Species**	**Growth condition**	**Doubling time^§^**	Replication time large chr*	Replication time small chr*	**Ori/ter ratio large chr^#^**	**Ori/ter ratio small chr^#^**
*V. para*	3%LB37°C	12–14 min	27.4 min	15.6 min	4.31	2.30
*V. para*	3%M937°C	50–60 min	27.4 min	15.6 min	1.41	1.22
*V. para*	3%LB20°C	36–42 min	27.4 min	15.6 min	-	-
*V. chol*	LB37°C	16–20 min	23.8 min	10.0 min	2.50	1.47
*V. vuln*	3%LB37°C	18–22 min	28.3 min	14.6 min	2.67	1.66

^§ ^Doubling times were determined for OD_600 _values around 0.5.

*Replication times were estimated considering a bidirectional replication speed of 1000 nt/s for both chromosomes of *V. parahaemolyticus*, *V. cholerae *and *V. vulnificus*

^#^Ratios were calculated using the average values for the estimated doubling times.

LB = Luria-Bertani medium containing 1% NaCl, 3%LB = Luria-Bertani medium containing 3% NaCl, 3%M9 = M9 medium containing 3% NaCl and 0.4% glucose.

Based on the established maximum replication fork movement of 1000 nt/s for *E. coli *[30], chromosome size can be used to estimate replication time. With an estimated replication time, knowledge about the doubling time enables predictions about origin/terminus (ori/ter) ratios for a replicon [21]. For this, we used the chromosome sizes given for the genomic strain of *V. parahaemolyticus *RIMD2210633, and employed pulsed-field gel electrophoresis to determine approximate sizes for the chromosomes of *V. cholerae *RIMD2203577 and *V. vulnificus *ATCC27562 (Table 1) before calculating ori/ter ratios for each separate chromosome (Table 2). The highest ratio (4.31) is estimated for the large chromosome of the quickly multiplying *V. parahaemolyticus*. These cells are also supposed to show relatively large inter-chromosomal difference in ori/ter ratios (4.31/2.30 = 1.87). For the large chromosomes of *V. cholerae *and *V. vulnificus*, the approximately equal ratios (2.50 and 2.67, respectively) reflect that the faster doubling time for the former is compensated by the larger size for the latter. Similarly, a relatively low ori/ter ratio for the small *V. cholerae *chromosome (1.47) is explained by its small size. Furthermore, the large size difference between the two *V. cholerae *chromosomes gives an inter-chromosomal ori/ter ratio (2.50/1.47 = 1.70) that is only slightly lower than for the much faster multiplying *V. parahaemolyticus *cells. Finally, a comparison between *V. parahaemolyticus *grown in rich and minimal media suggests that large variations in ori/ter ratios can be expected, especially for the large chromosome.

### Quantification of large and small chromosome origins and termini with RT-qPCR

**Figure 1:**
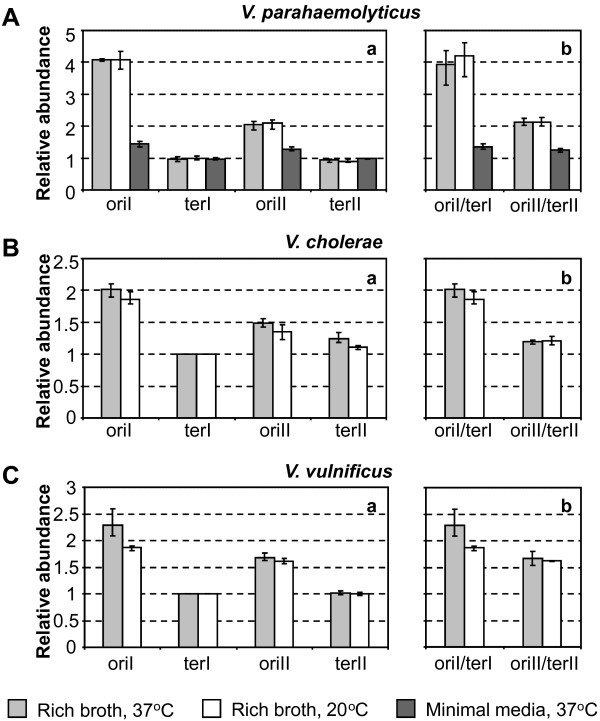
**Relative quantities of origin and termini proximate DNA in exponentially growing vibrios**. Relative amounts of origin and terminus proximate DNA from large (oriI and terI) and small (oriII and terII) chromosomes was determined with RT-qPCR for exponentially growing *V. parahaemolyticus *(Aa), *V. cholerae *(Ba) and *V. vulnificus *(Ca). Also shown are origin/terminus ratios for the separate chromosomes of *V. parahaemolyticus *(Ab), *V. cholerae *(Bb) and *V. vulnificus *(Cb). Bars display means for three or five experiments ± standard deviation and represent relative amounts of gDNA from cultures grown in rich broth at 37°C (light grey), from rich broth cultures at 20°C (white) and from cultures in minimal media grown at 37°C (dark grey). Large and small chromosome origin/terminus ratios were compared by paired t-tests assuming equal variances between groups and displayed significant differences (*P *< 0.05) for all strains and under all growth conditions.

To experimentally define origin and termini quantities and ratios, RT-qPCR was applied on genomic DNA (gDNA) using primers with target sites located near each origin and terminus of *V. parahaemolyticus*, *V. cholerae *and *V. vulnificus *(Additional file 1). As we wanted to determine relative target abundance in exponentially growing cells, gDNA from non-replicating cells with equal numbers of large and small chromosome origins and termini were used as reference samples (Additional file 2). As is significative for actively replicating chromosomes, the results indicate a relative increase in origin proximate DNA for both chromosomes in all species and under all growth conditions tested (Figure 1Aa, Ba and 1Ca). The figure also shows that whereas relative abundances of origin proximate DNA appear to be higher for the large *Vibrio *chromosomes than the small, there seem to be similar or only slightly different amounts of termini proximate DNA. In agreement with previous results from *V. cholerae *[15], this indicates a much earlier replication start for the large chromosome while termination for both chromosomes occurs within a short time span. Therefore, this suggests that such earlier replication start for the large chromosome could be a common trait for vibrios.

To better visualise gene dosage differences, ori/ter ratios were determined for each chromosome (Figure 1Ab, Bb and 1Cb), and in all instances the large chromosomes show a significantly higher ori/ter ratio than the small (*P *< 0.05). A comparison between *V. parahaemolyticus *grown in minimal and rich media at 37°C shows a much larger increase in gene dosage for the large than for the small chromosome (Figure 1Ab). This is consistent with doubling time based calculations (Table 2) and agrees with previous results from *V. cholerae *demonstrating that growth rate has a much larger impact on gene dosage for the large than for the small chromosome [15,16]. For low temperature cultures in rich media, however, there appear to be only minor differences in ori/ter ratios compared to rich media cultures grown at 37°C (Figure 1Ab, Bb and 1Cb). With consideration to the much faster doubling times at the higher temperature, this could suggest that replication is temporarily blocked or slowed in low temperature grown cells.

### Microarray based visualisation of replication dynamics

**Figure 2:**
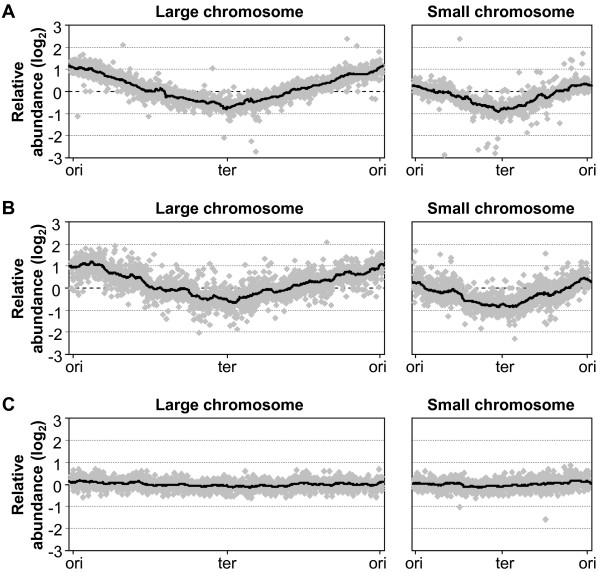
**Replication pattern for exponentially growing *V. parahaemolyticus *determined by gDNA/gDNA microarrays**. Genomic DNA (gDNA) from *V. parahaemolyticus *in exponential phase (OD_600 _~ 0.5) grown in LB with 3% NaCl at 37°C (A) or 20°C (B) or in M9 with 3% NaCl supplemented with 0.4% glucose (C) was compared against gDNA from non-replicating cells on microarrays spotted with DNA from all ORFs. Large and small chromosomes are linearised from the origins over the termini and back to the origins. Grey diamonds represent individual data points and black trend-lines show a sliding average for 50 data points. Scales on the x-axes are an approximate illustration of the respective lengths of the large and small chromosome.

A weakness in the above determinations is that they are built on assumptions about an equal and bi-directional replication rate for both chromosomes. To avoid this and get a more detailed and quantitative view of the replication dynamics, we next performed microarray analyses comparing gDNA from exponentially growing *V. parahaemolyticus *in rich media at 37 and 20°C and poor nutrient broth at 37°C against gDNA from non-replicating cells. The resulting replication patterns are shown in Figure 2 and display gene dosage as a decrease in DNA copy numbers when moving away from the origins of replication. For cells grown in rich broth at 37°C, smooth and similar slopes indicate an even replication progress, both within and between the two chromosomes, which lead to terminations at locations diametrically opposite to the origins of replication (Figure 2A). Also seen is an increase of nearly two orders of magnitude for origin over terminus proximate DNA quantities for the large chromosome, while a similar comparison for the small shows an increase of ~1.2 (Figure 2A). These values correspond to a large chromosome ori/ter ratio slightly below 4 and a small chromosome ori/ter ratio of ~2.3, which is in agreement with the RT-qPCR results (cf. with Figure 1Ab). Moreover, the replication patterns show a higher abundance of large over small chromosome origins while there are approximately equal amounts of termini for both chromosomes. Again, this indicates that termination rather than initiation occurs at a similar time in the cell cycle which is in agreement with previous results from *V. cholerae *[15]. For cells grown in rich media at 20°C (Figure 2B), ori/ter ratios and quantities are also confirmed (cf. with Figure 1A) and the overall replication patterns are very similar to those obtained for cells incubated at the higher temperature (cf. with Figure 2A). However, a closer comparison reveals a slightly more stuttered pattern which suggests that replication is temporarily arrested at 20°C. Although this could partly explain why large gene dosage differences are maintained despite longer doubling times, the relatively continuous decrease in DNA copy numbers emphasises slowed replication kinetics as the major contributor. Indeed, such temperature dependent change in replication speed has previously been detected in *E. coli *where cultures incubated at 14°C maintained similar replication time/doubling time ratios as cultures grown at 37°C [31]. Therefore, this suggests that the maintained gene dosage differences at a lower growth temperature are due to a slower replication kinetics that compensates for the less frequent initiation events. For cultures grown in minimal broth, only very small differences in DNA quantities along and between the chromosomes were seen and no clear locations for replication termination were discernible (Figure 2C). It therefore seems like the assay lack sensitivity for reliable determinations of replication dynamics for cells grown under these conditions. Nevertheless, the RT-qPCR results (Figure 1) and also previous analyses on *V. cholerae *cells grown in minimal media [15] imply a similar replication dynamics as for cells grown in rich broth.

Previous analyses have indicated a relatively fast replication progress for *V. cholerae *with replication fork movements around [16] or just below [15] 1000 nt/s for cells grown at 37°C. The slightly lower gene dosage differences for the RT-qPCR and microarray analyses compared to the doubling time based estimates (cf. Figure 1 and 2 with Table 2) indicate an average replication speed that may exceed 1000 nt/s. However, it must be considered that determination of a correct replication speed relies on a number of factors and inconsistencies may be due to (i) variations in doubling times estimates, (ii) differences in strains and growth media, (iii) errors in DNA target quantifications, (iv) errors caused by sampling handling and (v) the possibility that reference samples display a certain degree of replicating activity. Nevertheless, our results confirm a fast replication speed for vibrios. In addition, the replication speed seems slowed down to approximately one third for cells grown at 20°C as gene dosage differences were maintained (Figure 1 and 2) while doubling times increased threefold (Table 2).

### Microarray based examination of large and small chromosome expression

**Figure 3:**
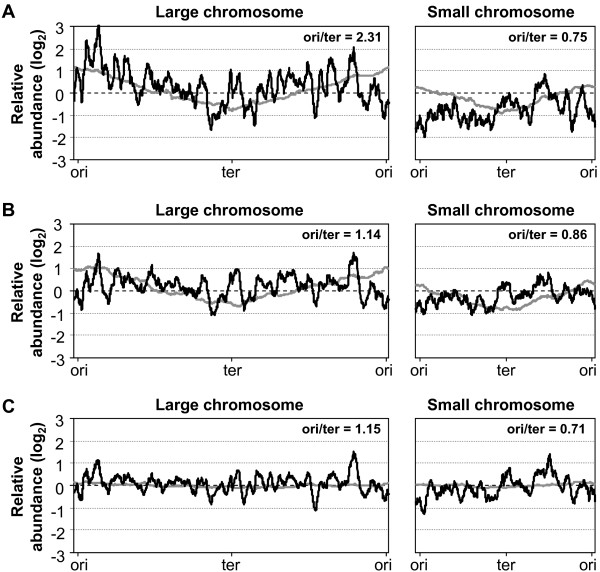
**Genome-wide expression in exponentially growing *V. parahaemolyticus *determined by cDNA/gDNA microarrays**. Gene expression was determined by comparing cDNA generated from cells in exponential phase (OD_600 _~ 0.5) grown in LB with 3% NaCl at 37°C (A) or 20°C (B) or in M9 with 3% NaCl supplemented with 0.4% glucose (C) against gDNA from non-replicating cells. Results are presented as black trend-lines that show sliding averages for 50 data points. Trend-lines from the previous gDNA quantifications (in grey) are included for comparison. Numbers display ori/ter ratios determined by linear regression analyses on expression data plotted against distances from the origin of replication. Scales on the x-axes display approximate chromosomal lengths.

The above analyses display rather large and growth dependent differences in gene copy numbers within and between the chromosomes. To examine whether this affect expression levels, we compared cDNA derived from *V. parahaemolyticus *grown in rich media at 37 and 20°C and poor nutrient broth at 37°C against gDNA from non-replicating cells. In addition, ori/ter ratios were determined by linear regression analyses on expression data sorted with respect to distances to the origins of replication. The resulting expression patterns and ori/ter values are shown in Figure 3 and to facilitate comparisons replication patterns are also displayed. As can be observed, the expression data displays very large variations in comparison to DNA quantities. Nevertheless, it is clearly distinguishable that average expression levels are higher from the large chromosome. Also in agreement with the replication patterns is that large chromosome expression levels appear to gradually decrease with an increased distance from the origin of replication, which is also supported by ori/ter ratios larger than 1. For the small chromosome, however, there appears to be no link between gene copy numbers and expression levels. Instead, general expression levels are low and ori/ter ratios indicate decreased rather than increased expression for origin-proximate relative to terminus-proximate genes. Therefore, this suggests that while gene expression from the large chromosome shows a tendency to follow gene dosage, expression from the small is more strictly regulated. A comparison between different growth environments lends further support for this statement in that large chromosome gene dosage effects are more pronounced for the fastest growing cells (cf. Figure 3A with 3B and 3C). Similarly, small chromosome expression seems even more tightly regulated when doubling times are shortened as DNA levels by far exceeds expression levels for cells grown in rich media at 37°C but not at a lower temperature or in minimal media (cf. Figure 3A with 3B and 3C). Also notable is that gene dosage effects are less pronounced for cells grown in rich media at a lower than a higher temperature although relative DNA quantities were comparable (cf. Figure 3A and 3B). This indicates that not only replication but also other cellular processes slow with a decreased temperature.

Although gene dosage and growth environment appear to have an influence on expression levels, the expression patterns also suggest other impacts. Several examinations have pointed out the presence of regularities in bacterial gene expression data where local expression maxima are found at periodicities of around [32] or slightly above [33,34] 100 kb. These patterns have been explained by higher order nucleoid structuring where the DNA is compacted into one or two large helices containing loops of approximately 100–120 kb lengths [32-34]. To examine whether periodicities are present in our expression data, grids were fitted to match local peaks. For both chromosomes periodic patterns of approximately 100 kb in length were detected (Additional file 3). Therefore, it appears like, in addition to gene dosage, also higher order chromosomal structuring has an influence on vibrio expression.

### Examination of genetic distribution within the *Vibrio *genome

The above experiments show that differing size and initiation timing creates higher average gene dosage and gene copy numbers on the large *Vibrio *chromosome. The results also show that expression levels from the large but not the small chromosome tend to follow gene dosage in a growth rate dependent manner. To gain a better understanding of these replication and expression patterns we next examined the distribution of different gene types within the *Vibrio *genome.

**Figure 4:**
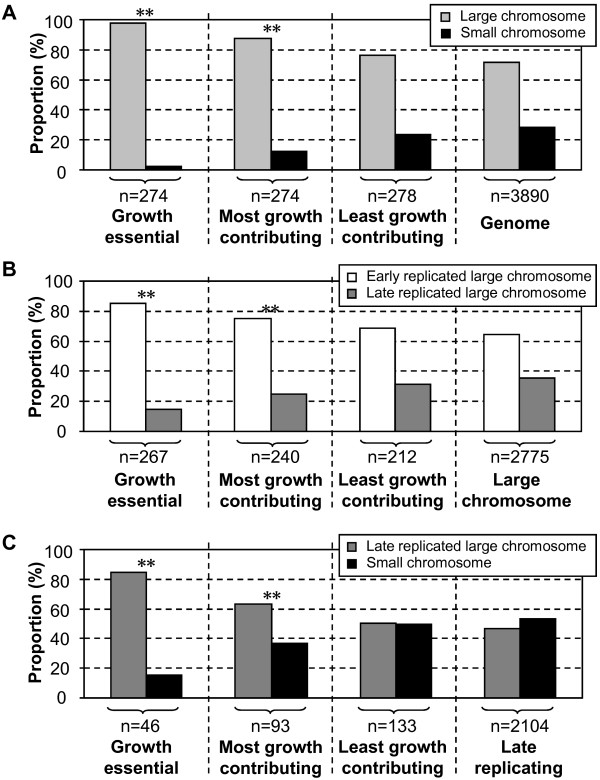
**Distribution of growth related genes within the *V. cholerae *genome**. Distributions of orthologs to growth essential, the most growth contributing and the least growth contributing *E. coli *genes between (A) the large and the small chromosome, (B) the origin- and the terminus-proximate part of the large chromosome and (C) the terminus-proximate part of the large chromosome and the small chromosome is shown together with distributions of all *V. cholerae *genes. Light grey bars represent the large chromosome, black bars the small chromosome, white bars the origin-proximate part of the large chromosome and dark grey bars the terminus-proximate part of the large chromosome. Sizes (n) of each group are shown and stars indicate that distributions are significantly different from an average determined by chi-square tests for comparison of two proportions (* *P *< 0.05, ** *P *< 0.01).

In a first analysis we wanted to see how genes related to growth were distributed within the genome. As comprehensive information about such genes is lacking for vibrios, we instead used data from a systematic knock-out analysis of all genes in the closely related bacterium *Escherichia coli *[35] and determined their closest orthologs in the *V. cholerae *genome using BLASTO [36]. Initially, we examined the distribution of orthologs to essential, the most growth contributing (genes whose absence create the largest growth defects for *E. coli *in LB) and the least growth contributing genes (*E. coli *genes that disturb growth in LB the least when absent) between the two chromosomes (see the Method section for a more detailed description). The results are displayed in Figure 4A and show a clear over-representation of orthologs to growth essential genes on the large chromosome. This is in agreement with findings from whole genome sequence analyses both for *V. cholerae *[11] and other *Vibrio *species [37-40], which have pointed out that many genes involved in essential biosynthetic pathways locate on the large chromosome, while few such genes are found on the small. For orthologs to genes that contribute the most to growth there was also a strong over-representation on the large chromosome while orthologs to the least growth contributing genes did not display a significant deviation from a random distribution between the chromosomes. Therefore, this analysis suggests a tendency where an increased importance for growth parallels a preferential location on the large chromosome. With consideration to the expression patterns this seems logical as only the large chromosome display growth rate dependent variations in expression levels.

We next analysed gene distribution between the early and late replicated parts of the large *V. cholerae *chromosome. The early replicated part was defined as being replicated before initiation of the small chromosome, assuming an equal bi-directional replication speed for both chromosomes and a synchronous termination. The results show that orthologs to both growth essential and the most growth contributing genes are over-represented within the early replicated part, while orthologs to the least growth contributing genes do not show a significantly biased distribution (Figure 4B). This distribution is in agreement with a previous notion that essential genes, and especially highly expressed such genes, show a clear tendency to locate near the origin of replication in *E. coli *[29]. The explanation given for this distribution was that the highly expressed essential genes benefit from a high gene dosage. With consideration to our expression results, this argument also seems suitable for vibrios. An additional benefit with an early replication for such genes in vibrios could be that this gives the cells more time to build up enough supplies of gene products required later in the cell cycle, including the additional metabolic burden of replicating the small chromosome [15]. It is also possible that an early replication of genes important for growth allow the cells to quickly adopt their expression to changes in growth conditions.

We also compared the distribution of orthologs to growth related genes between the late replicating part of the large chromosome and the small chromosome. Essential and most growth contributing genes were clearly under-represented on the small chromosome while genes contributing the least to growth appeared more randomly distributed (Figure 4C). This distribution contrasts with the observation that these two genome parts show a similar gene dosage (Figure 2). However, the disproportionally low number of growth important genes on the small chromosome agrees with the gene dosage and growth rate independent expression observed for this part of the genome (Figure 3). In summary, the results displayed in Figure 4 show that the distribution of genes central for growth is connected to replication timing and gene dosage effects.

**Figure 5:**
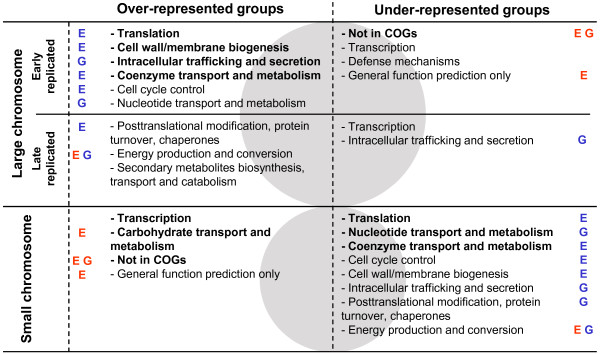
**Distribution of COGs within three different parts of the *Vibrio *genome**. Relative over- and under-representation of COGs within the origin-proximate (upper panel) and terminus-proximate (middle panel) parts of the large chromosome and within the small chromosome (lower panel) are shown. Categories listed show agreeing distributions within each of five *Vibrio *species (see Table 1) and categories in bold display distributions that are significantly different from an average in each separate species (*P *< 0.05, see Additional file 4). Blue tags represent an over-representation of essential (E) or most growth contributing (G) genes within the category while red tags represent an under-representation (see Additional file 5).

To extend the analysis of gene type distribution and attempt to better understand the expression patterns, we next sorted all *Vibrio *genes into different categories according to the classification system from Clusters of Orthologous Groups of proteins (COGs) [41]. Data available for five sequenced and annotated *Vibrio *genomes (Table 1) were used and relative over- and under-representation of 21 gene categories were examined within the early and late replicated parts of the large and within the small chromosome (see Methods and Additional file 4). Relative abundance of growth essential and most growth contributing genes within each category was also determined (see Methods and Additional file 5). The result is summarised in Figure 5 and display several distinct differences. In agreement with above observations, categories important for proliferation, such as "Translation", "Cell wall/membrane biogenesis", "Intracellular trafficking and secretion", Coenzyme transport and metabolism", "Cell cycle control" and "Nucleotide transport and metabolism" are over-represented on the early replicated part of the large chromosome and concurrently under-represented on the small. A missing category, however, is the "Replication, recombination and repair" genes that should benefit from an early replication and growth rate related gene dosage effects. Indeed, four of the five vibrios show an over-representation of this group on the early replicated part of the large chromosome and it is only *P. profundum *that, for unknown reasons, deviates from this pattern (Additional file 4). The categories over-represented on the small chromosome are generally of little importance for growth and several show a concurrent under-representation near the origin of replication of the large chromosome (Figure 5). A high abundance of undefined genes and genes lacking orthologs is in line with earlier observations from genome sequencing projects [11,37-40] and support speculations that the small chromosome could provide the bacteria with many species specific traits [11,42]. Furthermore, the over-representation of "Carbohydrate transport and metabolism" genes suggests that the small chromosome could be important for adaptation to different growth environments with varying energy sources, as was previously proposed [40]. Also striking is the high over-representation of "Transcription" genes and the concurrent under-representation on both parts of the large. To better understand this distribution it should be considered that transcription genes constitute a very heterogeneous group with regard to expression. It comprises both global regulators required at high concentrations to reach their many targets and much less expressed local regulators that co-localise with their targets either through direct proximity [43] or by three-dimensional proximity created by nucleoid structures [44]. This could mean that many transcriptional regulators on the small chromosome exert their action through co-localisation while global regulators gather on the large. Supporting this is that among the 20 transcriptional regulators that regulate the highest number of genes in *E. coli *[45], 18 have orthologs in *V. cholerae *and all of these locate on the large chromosome (data not shown). Therefore, a majority of the "Transcription" genes on the small chromosome are likely to regulate nearby genes. Furthermore, their high abundance could provide an explanation to why expression from this part of the genome is more strictly regulated and display independence from gene dosage.

## Discussion

Until recently it was believed that the two chromosomes of *V. cholerae *always initiated replication in a synchronous manner [14]. However, Rasmussen and co-workers introduced a new paradigm for *V. cholerae *replication dynamics when suggesting and confirming a model of termination synchrony [15]. Furthermore, they offered an explanation for why previous results had indicated initiation synchrony in that only large chromosome initiation is inhibited by treatment with the antibiotic rifampicin, which is commonly used prior to chromosome copy number determinations. The data shown in Figure 1 and 2 was obtained without antibiotics and confirm a model of termination synchrony. In addition, the results show that this model is applicable to additional *Vibrio *species and also to a differing rate of replication.

The generally high, partly gene dosage and growth rate dependent expression levels from the large chromosome is in agreement with vibrio replication dynamics. The high abundance of genes important for growth near the origin of replication of the large chromosome also makes sense as this provides both a fast and a powerful control of their expression in response to changed growth conditions. However, the gene dosage independent expression pattern for the small chromosome is more difficult to, at least directly, explain by replication dynamics. Instead, a high abundance of transcription related genes could be responsible for the more stringent expression regulation. Such tighter expression regulation is also in agreement with a disproportionally low abundance of growth important genes, even in comparison to the part of the large chromosome that is replicated at the same time. In addition, it is possible that the high abundance of species specific genes on the small chromosome further contributes to its low and gene dosage/growth rate independent expression levels. A support for this is that more newly acquired genes tend to be much more stringently regulated than genes with a long history within the genome [45,46].

Although the differences in gene dosage dependency between the two chromosomes may be explained by genetic distribution, a remaining question is why this genetic arrangement has evolved. One explanation could be that the lack/very low abundance of gene dosage depending genes on the small chromosome makes it more genetically flexible. Such flexibility could benefit the bacteria as a whole in that new genetic traits that improve the adaptive capacity are more easily gained [6]. Translocation of growth essential and growth contributing genes from the large to the small chromosome could decrease this flexibility which may explain why such genes are maintained on the large chromosome. An additional factor that likely restricts movement of growth important genes is the earlier replication start for the large chromosome. As we show here, a vast majority of growth essential and growth contributing genes locate on the origin-proximate part of the large chromosome. Translocation to any other part of the genome would delay their replication timing and decrease their dosage which could affect overall bacterial fitness. Indeed, support for a differential genetic flexibility is found in pair-wise comparisons of genetic content of different *Vibrio *species, which show a much higher variability between the small chromosomes [4,5]. In addition, these comparisons reveal a larger variability within the terminus-proximate than within the origin-proximate part of the large chromosome, which has also been noted by others [38,40]. Therefore, flexibility issues and, hence, adaptive advantages may, together with differing initiation timings, be responsible for the distinct genetic distribution and differential gene dosage dependency that appears to be maintained throughout the vibrio family.

With the above discussion in mind, it is interesting that genetic arrangements similar to that of vibrios are also found within other bacterial families. Inter-strain and inter-species comparisons of the alpha-3 subgroup proteobacteria *Rhodobacter sphaeroides *[47] and the alpha-2 subgroup *Brucella *family [48,49], respectively, display the presence of a large evolutionary conserved and a small fast evolving chromosome. Similarly, whole genome sequences of the beta-proteobacteria *Ralstonia solanacearum *[50] and *Burkholderia pseudomallei *[51] reveal the presence of a larger replicon that harbors most genes related to growth and survival and a smaller replicon that contain disproportionately high numbers of unclassified genes and genes related to transcription. Although replication dynamics and gene dosage effects have not been examined for these species, the strong genomic similarities with vibrios suggest that both of these factors could be influential. Moreover, the fact that a similar genomic structure appears to have arose more than once support the idea that it provides an important fitness advantage.

## Methods

### Bacterial strains and growth conditions

Bacterial strains used for experimental and computational analyses in this study are listed in Table 1. *V. parahaemolyticus*, *V. cholerae *and *V. vulnificus *from frozen glycerol stocks were grown in 2 ml cultures overnight at 20 or 37°C in either M9 media supplemented with 3% NaCl (w/v) and 0.4% glucose (3%M9), Luria-Bertani (LB) broth or LB containing 3% NaCl (3%LB). Cultures were diluted 1:1000 in 2 ml fresh media and incubated at 37 or 20°C with constant shaking at 210 rpm. At harvest, cultures were immediately transferred to – 30°C ice/NaCl/ethanol slurry followed by centrifugation (8000 g, 3 min, 4°C). To obtain non-replicating cells, overnight cultures were either plated onto LB agar followed by a 24 h incubation at 20°C and an additional 24 h incubation at 4°C or incubated in 2 ml liquid media (3%M9, LB or 3%LB) for 24 h at 20 or 37°C. Stationary phase liquid cultures were treated for 2 h with 20 μl rifampicin (50 mg/ml dissolved in DMSO) before harvest to finish ongoing replication rounds without initiating new ones.

### Pulsed-field gel electrophoresis

Estimates of chromosomal sizes were performed with pulsed-field gel electrophoresis (PFGE) on a CHEF Mapper XA system using reagents provided in the GenePath Univeral Module (Bio-Rad). Samples of *V. cholerae*, *V. vulnificus *and *V. parahaemolyticus *were prepared according to the manufacturers' instructions and loaded along with a molecular marker onto a 0.8% agarose gel prepared with and run in 1xTAE (40 mM Tris-acetate, 1 mM EDTA, pH 8.0) containing 500 μM thiourea [52]. The electrophoresis was run at 14°C for 48 h with a switch time of 500 s at 3 V/cm and an included angle of 106°.

### Determination of doubling times and estimations of replication times and origin/terminus ratios

Bacterial cultures were prepared as described above and samples were harvested every 10 minutes for dilution in series and plating onto LB plates for colony counts or every 30 minutes for OD-measurements. Doubling times were determined for mid-exponential phase cultures using the change in colony counts for a one hour time span around OD_600 _~ 0.5, by first calculating the number of generations (n):

$$n=\frac{\log b-\log B}{\log2}$$

Here, b is the number of CFU at the end and B the number of CFU at the beginning of the one hour time interval. Doubling times (τ) were given by dividing the time interval (t) with the number of generations (n):

$$\tau =\frac{t}{n}$$

To estimate the time required to complete one replication round (C) for each chromosome, a bidirectional replication speed of 1000 nt/s was used:

$$\text{C}=\frac{Chromosome\;size}{2\times 1000\times 60}$$

Given that initiation of replication occurs simultaneously for all origins on a replicon [53] it can be assumed that every new required round of replication results in a doubling of the origin/terminus ratio (r_O/T_). The following formula, taken from [21], was used to calculate (r_O/T_):

r_O/T _= 2^C/τ^

Calculations are summarised in Table 2.

### Extraction of nucleic acids

Genomic DNA (gDNA) was extracted with DNeasy^® ^Tissue Kit (Qiagen) following the manufacturer's instructions including a prolonged RNase treatment step (15 minutes) and was finally eluted in 2 × 100 μl AE buffer. DNA samples were further purified by addition of equal volumes of phenol and chloroform with subsequent phase separation by centrifugation before precipitation with 1/10 volume 3 M CH_3_COONa (pH 5.2) and 2.6 volumes ethanol. DNA was pelleted by centrifugation and further washed with 1 ml 70% ethanol before being dissolved in H_2_O.

Total RNA was extracted from bacterial pellets with 1 ml TRIzol^® ^Reagent (Invitrogen) following the manufacturer's protocol. RNA pellets were re-suspended and treated with DNase I according to instructions that accompany the RNase-free DNaseI set (Qiagen) before purification, precipitation and wash with phenol/chloroform, 3 M CH_3_COONa (pH 5.2)/ethanol and 70% ethanol, respectively. RNA was re-dissolved in 100 μl H_2_O and further purified using RNeasy^® ^Mini Kit (Qiagen) before elution in H_2_O. RNA was subjected to a further precipitation (3 M CH_3_COONa (pH 5.2)/ethanol) and wash (70% ethanol) before finally being dissolved in H_2_O.

### RT-qPCR

Relative quantification of origins and termini for both *Vibrio *chromosomes was carried out with either 10 ng (for *V. parahaemolyticus*) or 2.5 ng (for *V. cholerae *and *V. vulnificus*) gDNA with real time quantitative polymerase chain reaction (RT-qPCR). Reaction volumes were 20 μl and also included 1× Power SYBR^® ^Green PCR Master Mix (Applied Biosystems) and either of the primer pairs listed in Additional file 1. Importantly, primer pairs were selected to target stably integrated regions not thought capable to excise from their respective places on the genomes. An exception was a primer pair targeting a phage like element present at both *V. parahaemolyticus *termini that was selected to provide an additional reference for this species. As we aimed to quantify relative numbers of large and small chromosome origins and termini in exponentially growing cultures, reference samples with known relative quantities of these regions were required. To provide such references, gDNA was extracted from non-replicating cells that were pre-grown on LB-plates and incubated for 24 h at 4°C. To verify an equal relationship between large and small chromosome origin and termini, the samples were compared against gDNA from exponentially grown cells along with gDNA from non-replicating cells from liquid media cultures grown under two (*V. vulnificus*) or three (*V. parahaemolyticus *and *V. cholerae*) different conditions (see "Bacterial strains and growth conditions"). Similar ratios between origins and termini were obtained for all non-replicating samples, which confirmed their reliability as reference samples (Additional file 2). In addition, a pyrosequencing based analysis with a 20 fold coverage of the whole genome of *V. parahaemolyticus *confirmed approximately equal numbers of small and large chromosomes for stationary phase cells in 3%LB (unpublished data from the lab). Amplification efficiencies were validated with gDNA from both non-replicating (three separate experiments with five replicates) and exponentially grown cells (one experiment with five replicates) over a range of template concentrations (2.5–40 ng for *V. parahaemolyticus *and 0.625–10 ng for *V. cholerae *and *V. vulnificus*). Near linear dose responses and similar amplification efficiencies (89–100% for *V. parahaemolyticus*, 97–104% for *V. cholerae*, and 98–103% for *V. vulnificus*) were obtained by analysis with the 2^-ΔΔCT ^method [54] and indicated that reliable comparisons between the targets could be performed. Experimental analyses were performed in five double samples on gDNA extracted at three or five different occasions.

### Preparation of aminoallyl-labelled nucleic acids derived from gDNA and RNA

For generation of aminoallyl-labelled product from gDNA, the procedures described in [55] were followed. For generation of aminoallyl-labelled product from total RNA we used reagents included in SuperScript™ III Reverse Transcriptase Kit (Invitrogen). In brief, 20 μg RNA was mixed with 10 μg random hexamers in a 22 μl reaction that was incubated at 70°C for 5 min before cooling on ice. Addition of 5× First-Strand Buffer (8 μl), 0.1 M DTT (2 μl), SuperScript™ III RT (4 μl) and 10× dNTP-aminoallyl dUTP (4 μl) was followed by a 3 h incubation at 46°C. Samples were precipitated and washed as described earlier.

### Coupling with Cy3 and Cy5 dyes, hybridisation onto microarray slides and scanning

Coupling of aminoallyl-labelled products with Cy3 or Cy5 monofunctional reactive dyes was performed as described previously [55]. Microarray slides and hybridisation procedures are also described in [55], except that human CotI DNA (Invitrogen) replaced yeast tRNA and incubation/hybridisation temperatures were 55°C instead of 60°C. Fluorescence signals were measured and analysed according to previous descriptions [55]. Microarray data was submitted to Gene Expression Omnibus (GEO) [56] and has the serial number GSE9968.

### Computational analyses

To examine the distribution of essential and growth related genes in the *Vibrio *genome, we used growth and essentiality data from a genome-wide single gene knock-out study performed in *E. coli *[35] and deduced the nearest orthologs in *V. cholerae*. *E. coli *genes were classified into three groups; one containing all essential genes, a second (most growth contributing genes) containing the genes for which knock-outs displayed the slowest growth rates in LB media (OD_600 _< 0.604, see [35]), and a third (least growth contributing genes) comprising genes for which knock-outs displays the highest growth rates in LB media (OD_600 _> 0.823, see [35]). Next, protein sequences for the essential, most growth contributing and least growth contributing *E. coli *genes were searched against the NCBI COG database in BLASTO [36] using the default settings (E = 0.001, BLOSUM62). Best hits were sorted into three parts of the *V. cholerae *genome; an early replicated part of the large chromosome (defined as the part being replicated before initiation of the small chromosome assuming an equal bidirectional replication speed and a simultaneous replication termination), a late replicated part of the large chromosome (the part of the large chromosome that is not defined as being early replicated) and the small chromosome. Deviations from an average distribution were determined by comparison to the total number of genes within the different parts of the genome and significance levels were determined by chi-square tests for comparison of two proportions.

To examine the distribution of different gene categories, the classification system from Clusters of Orthologous Groups of proteins (COGs) [41] was employed. Genes belonging to 21 functional categories were counted within the early and late replicated parts of the large and the small chromosome of five *Vibrionaceae *species (see Table 1). Relative over- and under-representation was determined by comparing the size of each group against an average distribution of COGs within each genome part. Significance levels for deviations from average distributions were determined by chi-square tests for comparison of two proportions. Categories showing a similar deviation from an average in a specific genome part for all *Vibrionaceae *species were considered over- or under-represented and categories with a similar and significant deviation for each species were considered highly over- or under-represented.

To examine the relative abundance of growth essential or highly growth contributing genes within the separate COG categories, we first determined the number of essential and highly growth contributing genes for each COG. Proportions of essential genes within each category were compared to the proportion of essential genes in the whole genome while proportions of highly growth contributing genes among the non-essential genes in each category were compared to the proportion highly growth contributing among all non-essential genes. Significance levels for deviations were determined by chi-square tests for comparison of two proportions.

## Authors' contributions

RD and TI conceived and designed the experiments. RD performed most experiments and computational analysis. KI performed or assisted RD in some microarray experiments. RD wrote the paper with help from KI, TH and TI. TH and TI contributed reagents/materials/analysis tools.

## Supplementary Material

Additional file 1 **RT-qPCR primers with target positions**. The table shows sequences and target positions for RT-qPCR primers. Click here for file

Additional file 2 **Comparison of gDNA from non-replicating cell samples against gDNA from exponentially growing cells**. The figure shows relative amounts of origin and terminus proximate DNA in differently produced non-replicating samples of *V. parahaemolyticus*, *V. cholerae*, and *V. vulnificus *in comparison to exponentially growing cell samples. Click here for file

Additional file 3 **Periodic expression patterns from the large and small chromosome of V. parahaemolyticus**. The figure indicates periodicities in expression levels along both chromosomes of *V. parahaemolyticus*. Click here for file

Additional file 4 **Distribution of COG classified genes within three different parts of five Vibrionaceae genomes**. The table shows COG sizes within the early and late replicated parts of the large chromosome and the small chromosome in five *Vibrionaceae *species. Click here for file

Additional file 5 **COG classification of all, essential and highly growth contributing E. coli genes**. The table shows the distribution of all, growth essential and highly growth contributing *E. coli *genes within each of 21 COG categories. Click here for file
