# Supplementary material for: Differential replication dynamics for large and small Vibrio chromosomes affect gene dosage, expression and location
Source: BMC Genomics. 2008 Nov 26;9:559. doi: 10.1186/1471-2164-9-559 (PMC2612033; doi:10.1186/1471-2164-9-559)
Supplement: Additional file 1 — RT-qPCR primers with target positions. The table shows sequences and target positions for RT-qPCR primers. [file 1471-2164-9-559-S1.pdf]

Additional file 1 - RT-qPCR primers with target positions

Name	Sequence (5' -> 3')	Target position
VP oriI Fw	TGTTGCGCTTCTAGCACTGAGA	3287799-3287820
VP oriI Rev	CGCTTACGTGAGTTGCCATTC	3287899-3287879
VP terI Fw	TGAAATTGGCGAAGGTGGAA	1644714-1644733
VP terI Rev	TGCGGCTCTTTCGTGAAGAT	1644819-1644800
VP oriII Fw	GGCAACAGCACGTTCTTGTGTA	1876515-1876536
VP oriII Rev	TGGCTGAGCACGACAACATTTA	1876620-1876599
VP terII Fw	CAGCCGTAATTTGACCGGTAAA	956302-956323
VP terII Rev	AGCCTTGGATGCACTTTCTTCA	956405-956384
VP ter Fw	GTTGGCACTAATTGGCATCGA	1673680-1673700[#]<br>939300-939320[§]
VP ter Rev*	GCTCGTTCTGCTTCTGGTTTGT	1673781-1673760[#]<br>939401-939380[§]
VC oriI Fw	CAGGTGAACCAGCAAAATCGA	2959190-2959210
VC oriI Rev	TGGTATTGAAGCTCAATGCGG	2959290-2959270
VC terI Fw	TTCAAGCTGAGGCGGATTTG	1503396-1503415
VC terI Rev	GCTCATTGGCTTCTTGTGCTTC	1503500-1503479
VC oriII Fw	GCAGGCAATCGCTTTGCTT	3636-3654
VC oriII Rev	CATAACGATTGTGCTGCCGTC	3743-3723
VC terII Fw	CGCACAGCCTCAGCAAAAA	501354-501372
VC terII Rev	CCACGGCTAAACCAACCAGTT	501454-501434
VV oriI Fw	ACCACCTCGTTGGATAAGCCTT	3352630-3352651
VV oriI Rev	GGCTACATCAAGCGTCAGCAA	3352742-3352722
VV terI Fw	ACCAGCTAGAAAACATGCCGC	1736050-1736070
VV terI Rev	CCAAGCAGCCATTACAAGGAAA	1736154-1736133
VV oriII Fw	CGCGTTTCCATCAATGCTTT	1855713-1855732
VV oriII Rev	GGCTGAATCAACCCAACAGGT	1855817-1855797
VV terII Fw	GCAAGTCCGGCCAGTTTTTT	905810-905829
VV terII Rev	TGCGCCACCAATTCAAGTTT	905923-905904

*Targets the terminus region of the large chromosome with a mismatch at the sixth base
[#]Target position at the large chromosome
[§]Target position at the small chromosome
VP=*V. parahaemolyticus*, VC=*V cholerae*, VV=*V. vulnificus*
