# Supplementary material for: Differential replication dynamics for large and small Vibrio chromosomes affect gene dosage, expression and location
Source: BMC Genomics. 2008 Nov 26;9:559. doi: 10.1186/1471-2164-9-559 (PMC2612033; doi:10.1186/1471-2164-9-559)
Supplement: Additional file 2 — Comparison of gDNA from non-replicating cell samples against gDNA from exponentially growing cells. The figure shows relative amounts of origin and terminus proximate DNA in differently produced non-replicating samples of V. parahaemolyticus, V. cholerae, and V. vulnificus in comparison to exponentially growing cell samples. [file 1471-2164-9-559-S2.pdf]

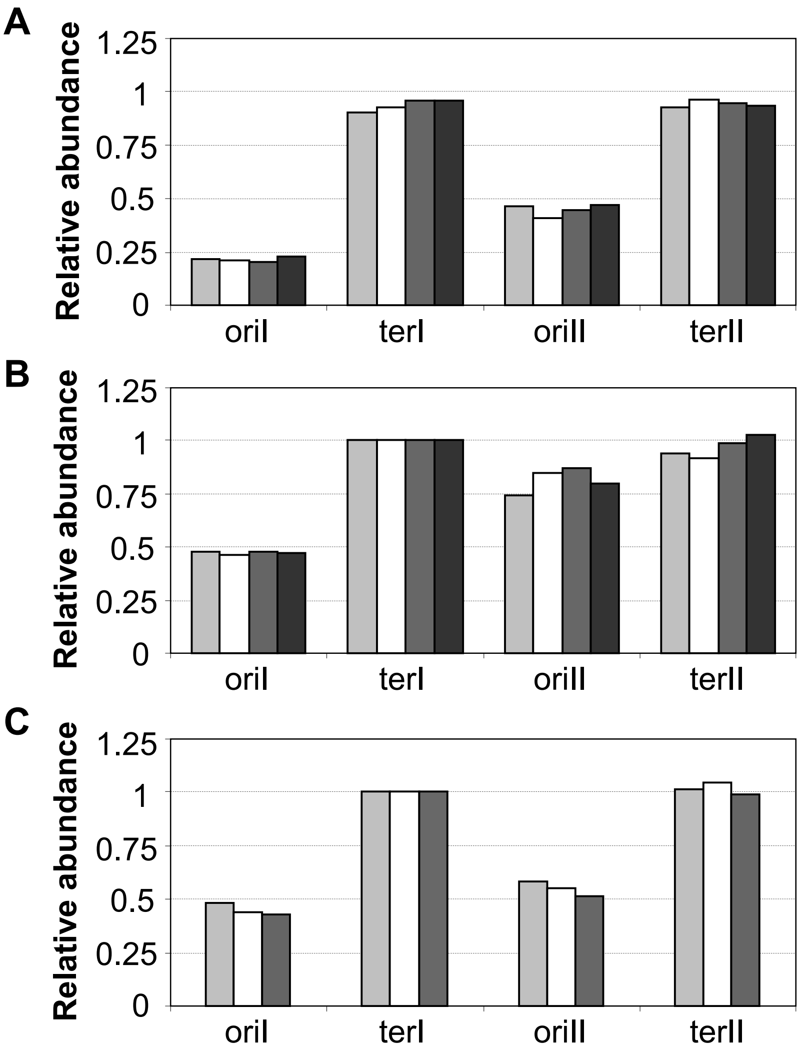

Additional file 2 - Comparison of gDNA from non-replicating cell samples against gDNA from exponentially growing cells

Relative amounts of origin and terminus proximate gDNA was determined for non-replicating *V. parahaemolyticus* **(A)**, *V. cholerae* **(B)**, and *V. vulnificus* **(C)** with RT-qPCR using gDNA from exponentially growing cells as references samples. Light grey bars represent gDNA from cells incubated on plate at 4°C for 24 h; white bars represent gDNA from cells grown until stationary phase in rich media at 37°C before addition of rifampicin (500 µg/ml final conc) and a further 2 h incubation; dark grey bars represents gDNA from similarly treated cells grown at 20°C and black bars represent gDNA from cells grown until stationary phase in minimal media at 20°C (*V. parahaemolyticus*) or 37°C (*V. cholerae*) before the rifampicin treatment. Experiments were performed with five replicates.
