# Supplementary material for: Differential replication dynamics for large and small Vibrio chromosomes affect gene dosage, expression and location
Source: BMC Genomics. 2008 Nov 26;9:559. doi: 10.1186/1471-2164-9-559 (PMC2612033; doi:10.1186/1471-2164-9-559)
Supplement: Additional file 3 — Periodic expression patterns from the large and small chromosome of V. parahaemolyticus. The figure indicates periodicities in expression levels along both chromosomes of V. parahaemolyticus. [file 1471-2164-9-559-S3.pdf]

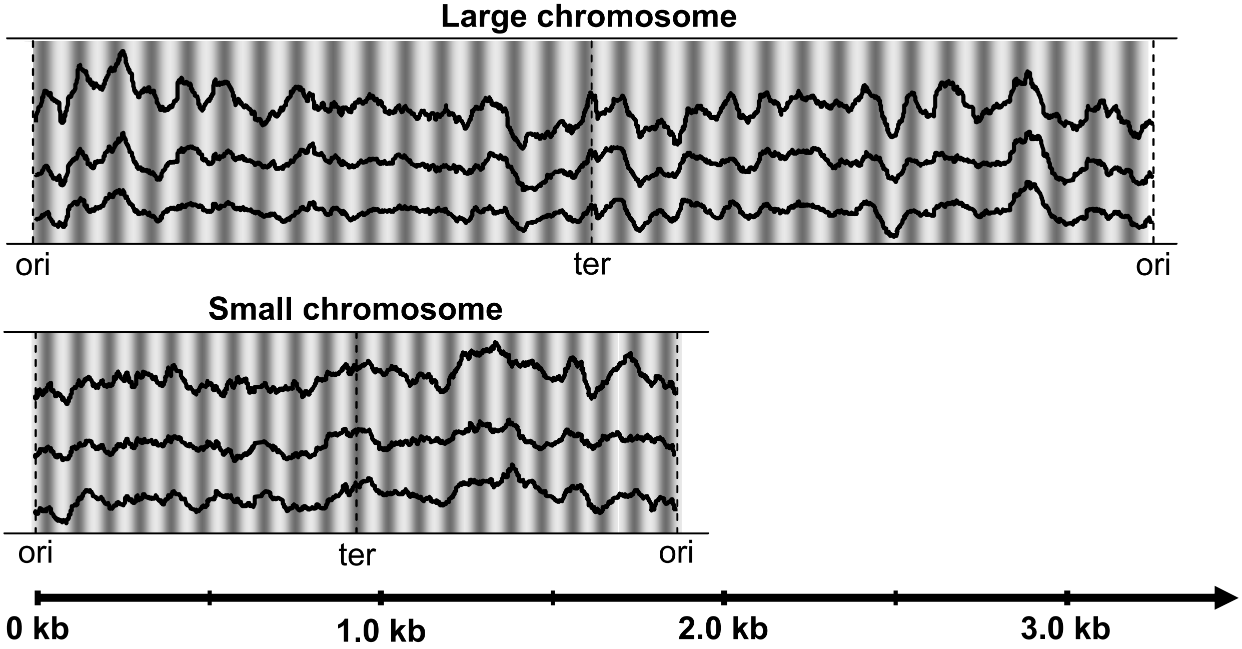

Additional file 3 - Periodic expression patterns from the large and small chromosome of *V. parahaemolyticus*

Gene expression patterns for the large (upper panel) and the small chromosome (lower panel) taken from Figure 3 are aligned and grids with equally distanced bars were manually fitted to local expression maxima along the chromosomes. A grid with a 107 kb distances between the bars showed the best fit for the large chromosome while a grid with 96 kb distances better reflected local expression maxima on the small.
