# Supplementary material for: Differential replication dynamics for large and small Vibrio chromosomes affect gene dosage, expression and location
Source: BMC Genomics. 2008 Nov 26;9:559. doi: 10.1186/1471-2164-9-559 (PMC2612033; doi:10.1186/1471-2164-9-559)
Supplement: Additional file 4 — Distribution of COG classified genes within three different parts of five Vibrionaceae genomes. The table shows COG sizes within the early and late replicated parts of the large chromosome and the small chromosome in five Vibrionaceae species. [file 1471-2164-9-559-S4.pdf]

Additional file 4 - Distribution of COG classified genes within three different parts of five *Vibrionaceae* genomes

	Early replicated part of large chromosome						Late replicated part of large chromosome						Small chromosome					
COG	VP	VC	VV	VF	PP	ALL	VP	VC	VV	VF	PP	ALL	VP	VC	VV	VF	PP	ALL
J	107**	142**	107**	125**	126**	607**	70	30**	79	60	53**	292**	24**	24**	28**	18**	35**	129**
K	90	109	97	99*	109	504**	105**	68	95**	78*	117**	463**	178**	94*	175**	135**	184**	766**
L	68**	88	91**	73*	117	437**	80	48	99	53	138	418	39**	42	45**	37*	190**	353**
D	14	22	18*	21	16	91**	18	10	19	14	20	81	5**	9	3**	11	9*	37**
V	15	14*	16	11**	15*	71**	26	24**	26	20	37	133*	29	11	30	32**	38	140*
T	65	92**	76**	64**	85	382**	106	98**	126	78	106	514	129*	93	160**	122**	101	605**
M	84*	102**	93**	121**	106**	506**	95	50	78	54**	95	372	72**	29**	59**	67	60**	287**
N	32	86**	32*	38*	72**	260	69	26	79**	60**	63	297**	59	27*	44	35	23**	188**
U	55**	72**	55**	63**	64**	309**	48	16	41	26	39*	170**	42*	6**	19**	22**	43	132**
O	46	75	43	59	61	284	75	37	75**	67**	77*	331**	56	29*	42*	27**	42**	196**
C	66	95	68	72	85	386	94	51	80	75	113	413*	77	44	67	54	101	343**
G	57	71**	78	80	62**	348**	58**	60	70**	40**	98	326**	117**	70*	162**	86	165**	600**
E	109	130	116	123	128	606	142	88	129	105	156	620	140	85	137	96	137	595
F	29	51**	29	40	39	188**	44*	14	42*	33	50*	183**	18**	12*	18**	12**	26**	86**
H	68**	94**	71**	79**	85**	397**	57	42	59	46	54**	258	31**	15**	25**	22**	29**	122**
I	22*	45	23	33	31	154*	56	21	53*	43**	52	225**	50	17	34	12**	54	167
P	67	91	69	72	75	374	60**	34*	65**	56	86	301**	120**	64	95*	68	86	433**
Q	9**	35	11**	10**	17**	82**	46**	20	40	28**	39	173**	30	12	36	19	51*	148
R	141	176	145	150	158	770	191	97	183	147	214	832	204	140*	198	142	217	901*
S	92	123	90	100	143**	548	142	82	124	107**	119	574	131	87	123	72*	115**	528
-	218**	295**	270**	168**	164**	1115**	429	202	509**	161**	409	1710	470**	339**	485**	281**	554**	2129**
Sum	1454	2008	1598	1601	1758	8419	2011	1118	2071	1351	2135	8686	2021	1249	1985	1370	2260	8885

Blue digits describe an over-representation and red digits an under-representation and stars indicate significance levels for biases from an average distribution determined by chi-square tests for comparison of two proportions (*$P<0.05$, **$P<0.01$)

J = Translation, K = Transcription, L = Replication, recombination and repair, D = Cell cycle control, V = Defense mechanisms, T =Signal transduction mechanisms, M = Cell wall/membrane biogenesis, N = Cell motility, U = Intracellular trafficking and secretion, O = Posttranslational modification, protein turnover, chaperons, C = Energy production and conversion, G = Carbohydrate transport and metabolism, E = Amino acid transport and metabolism, F = Nucleotide transport and metabolism, H = Coenzyme transport and metabolism, I = Lipid transport and metabolism, P = Inorganic ion transport and metabolism, Q = Secondary metabolites biosynthesis, transport and catabolism, R = General function prediction only, S = Function unknown, - = Not in COGs. VP = *V. parahaemolyticus*, VC = *V. cholerae*, VV = *V. vulnificus*, VF = *V. fischeri*, PP = *P. profundum*, ALL = values for all species combined.
