# Supplementary material for: Differential replication dynamics for large and small Vibrio chromosomes affect gene dosage, expression and location
Source: BMC Genomics. 2008 Nov 26;9:559. doi: 10.1186/1471-2164-9-559 (PMC2612033; doi:10.1186/1471-2164-9-559)
Supplement: Additional file 5 — COG classification of all, essential and highly growth contributing E. coli genes. The table shows the distribution of all, growth essential and highly growth contributing E. coli genes within each of 21 COG categories. [file 1471-2164-9-559-S5.pdf]

Additional file 5 - COG classification of all, essential and highly growth contributing *E. coli* genes

COG	Total number	Essential	Highly growth contributing
Translation	152	56**	15
Transcription	269	14	17
Replication, recombination and repair	225	15	15
Cell cycle control	29	15**	0
Defense mechanisms	46	2	3
Signal transduction mechanisms	158	5	13
Cell wall/membrane biogenesis	216	34**	19
Cell motility	103	0*	38**
Intracellular trafficking and secretion	117	10	23**
Posttranslational modification, protein turnover, chaperons	125	7	19*
Energy production and conversion	277	6**	40**
Carbohydrate transport and metabolism	269	7**	22
Amino acid transport and metabolism	329	7**	23
Nucleotide transport and metabolism	81	10	18**
Coenzyme transport and metabolism	128	24**	13
Lipid transport and metabolism	97	22**	3
Inorganic ion transport and metabolism	200	0**	8*
Secondary metabolites biosynthesis, transport and catabolism	65	4	2
General function prediction only	397	14*	27
Function unknown	209	5*	8*
Not in COGs	1192	60**	82**
All	**4684**	**317**	**408**

Blue digits describe an over-representation, red digits an under-representation and stars indicate significance levels for biases from an average distribution determined by chi-square tests for comparison of two proportions (**P*<0.05, ***P*<0.01)
